# Multifunctional Programmable Transmissive Metasurface with Phase and Amplitude Manipulation Capability

**DOI:** 10.1002/advs.202518176

**Published:** 2025-12-22

**Authors:** Hao Tian Shi, Rui Yuan Wu, Shi He, Xue Yun Guo, Xiao Qing Chen, Yi Ning Zheng, Bing Bing Zhu, Lei Zhang, Tie Jun Cui

**Affiliations:** ^1^ State Key Laboratory of Millimeter Waves Southeast University Nanjing China; ^2^ College of Information Science and Engineering Hohai University Nanjing China

**Keywords:** beamforming, hologram imaging, orbital angular momentum, phase‐amplitude joint control, programmable metasurface

## Abstract

Digital and programmable metasurfaces provide novel approaches to connect electromagnetic (EM) physics with information science for flexible EM wave manipulation and information modulation. However, most recent studies on programmable metasurfaces have been focused on reflective phase coding and only provide limited regulation capability. To overcome these limitations, we propose a transmissive programmable metasurface with both amplitude and phase coding. The function of the metasurface can vary according to different control voltages. Typically, the metasurface achieves multiple‐bit phase control and phase‐amplitude joint control. To prove the unique features, we design and fabricate a prototype of the metasurface to verify the functions of vortex beam generation, hologram imaging, and beam forming. The measured results show good performance of the proposed metasurface, implying powerful EM manipulation capability and indicating a wide range of application potentials in imaging, data storage, wireless communications, and so on.

## Graphical abstract

This work provides a multifunctional programmable transmissive metasurface with phase and amplitude manipulation capability in the sub‐6G band. By applying different coding strategies, the proposed metasurface can realize vortex beam generation, holographic imaging under phase coding mode, and multi‐beam forming in phase‐amplitude joint coding mode.

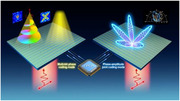

## 1 Introduction

With the advantages of simple structure, low power consumption, and easy integration, metasurfaces, as the 2D form of metamaterials, have drawn increasing attention from researchers in recent years [1, 2, 3, 4]. Unlike natural materials, metasurfaces are composed of artificially designed microstructures, making it possible to achieve many extraordinary phenomena, such as anomalous refraction and reflection [5], Vortex wave generation [6], and other complex beam regulation [7]. Due to these incredible electromagnetic (EM) wave manipulation abilities [8, 9, 10], metasurfaces are widely used in communication, sensing, and the Internet of Things (IoT) systems [11, 12, 13, 14]. Despite great progress in research on EM manipulation devices based on metasurfaces, including polarization conversion, meta‐lens, holographic imaging, and EM stealth [15, 16, 17, 18], the functions of traditional metasurfaces in these studies are fixed after fabrication. To solve this shortage, in 2014, Cui. et al., proposed a programmable metasurface [19], providing a possible solution to manipulate EM waves and change the functions of a given metasurface in real‐time. Besides EM wave manipulations, digital and programmable metasurfaces provide a tool to bridge the physical world and information systems, making it possible to encode and process EM information directly in the physical world, resulting in the concept of information metasurface and novel intelligent EM devices [20, 21, 22, 23].

Though many appealing studies have been carried out based on metasurfaces [24, 25, 26, 27, 28, 29], the EM manipulation ability of the metasurface is still limited. Most recent studies on programmable metasurfaces are limited by either phase manipulation ability [24, 25, 26] with only 1‐bit or 2‐bit phase coding states hindering the elaborate regulation of the EM field. Despite plenty of remarkable research achievements based on reflective metasurfaces [27, 28, 29], transmissive metasurfaces have the advantages of no occlusion, low profile, and easy integration, leading to broader application prospects. However, research on programmable transmissive metasurfaces [30, 31, 32, 33, 34] is limited by the shortcomings of complex structure, limited control ability, and low transmission efficiency [35, 36, 37]. With the growing demands of multifunctional and precise manipulation of the EM wave, joint manipulation of phase and amplitude [38, 39, 40, 41] has become an important research direction. Therefore, to fully develop the potential EM manipulation ability of metasurface and expand the application in dynamic EM waves control, mobile communication, and signal processing, it is essential to develop a novel programmable transmissive metasurface providing both phase and amplitude manipulation capability with a simple structure.

**FIGURE 1:**
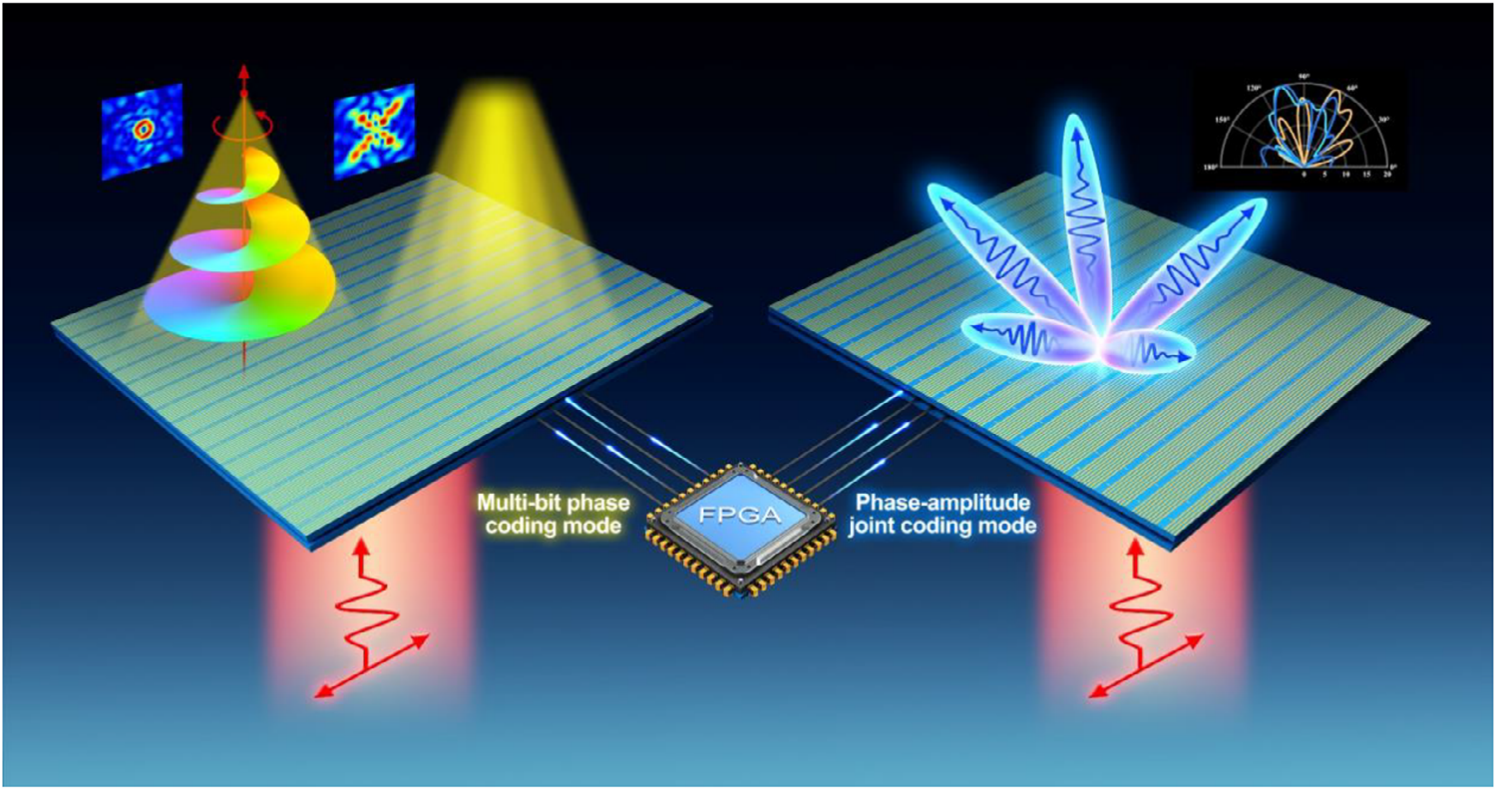
Schematic of the proposed metasurface. The Micro Control Unit, for example FPGA, controls the polarization conversion metasurface, providing two coding modes, the multi‐bit phase coding mode and the phase‐amplitude joint coding mode, to manipulate the EM wave.

To meet our design objective, a multifunctional transmissive metasurface is proposed, providing a solution to multi‐bit phase control and phase‐amplitude joint control systems. The structure and functions of the metasurface are illustrated in Figure 1. Inspired by the polarization conversion metasurface using Pancharatnam‐Berry (PB) phase to manipulate the polarization states and phase response [42, 43, 44, 45], we propose a novel metasurface with the ability of phase‐amplitude manipulation by fully developing the EM manipulation potential of polarization‐conversion metasurfaces, where transmissive phase can be manipulated through PB phase and structure phase and transmissive efficiency can be manipulated through polarization conversion efficiency. By applying different DC voltages, the varactors embedded in the metasurface will exhibit different capacitance values, regulating the phase and amplitude of the transmission EM wave. Through careful design of DC bias networks, each pixel of this metasurface can be controlled independently. To validate, simulations and experiments have demonstrated the typical applications in multi‐beam forming through phase‐amplitude joint manipulation and orbital angular momentum beam generation and near‐field imaging through phase‐only modulation. Compared with previous studies on the transmissive metasurface [35, 46, 47, 48, 49], the proposed metasurface has a simple structure, which makes it easy to fabricate. Besides, the low power consumption due to the varactors makes the proposed metasurface satisfy the demand for low‐power EM regulation. Furthermore, the polarization conversion feature enables the metasurface to reduce the interference of transmitted waves and diffraction waves, resulting in excellent performance in holographic imaging and OAM beam generation. Therefore, the proposed high‐performance multifunctional programmable metasurface in sub‐6 GHz shows promising applications in imaging, wireless communication, sensing, holography, and other scenarios.

## 2 Design of Metasurface

### 2.1 Design and Analysis of the Meta‐Particle

**FIGURE 2:**
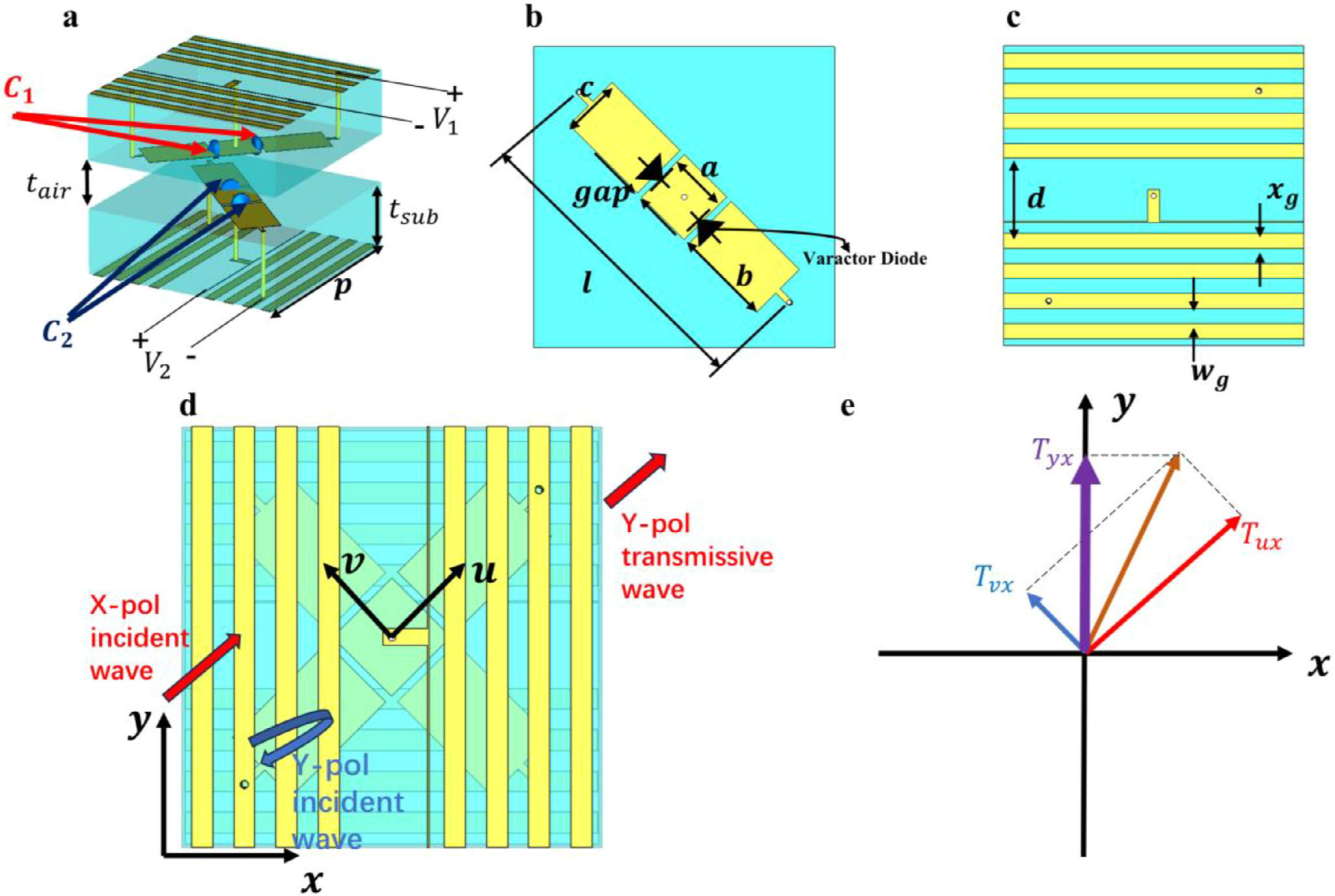
(a) The structure of the meta‐particles. (b) The top view of the board that constitutes the meta‐particle. (c) The bottom view of the board that constitutes the meta‐particle. (d) The schematic of the working principle of the meta‐particle. (e) The schematic of the principle of the amplitude and phase manipulation.

A conventional programmable metasurface based on a single EM resonant structure can usually be equivalent to a first‐order resonant circuit, which means that the phase response of the metasurface can only cover 180°. Though adding complexity to the metasurface structure can result in a wider phase coverage range, it will deteriorate the bandwidth and stability of the metasurface. To overcome this challenge, the proposed meta‐particle consists of two opposed structures that are rotated 90°. The proposed metasurface uses varactors to regulate the resonant state of the metasurface and extend the transmissive phase range from 180° to 360° through PB phase, while manipulating the transmissive amplitude according to different polarization conversion efficiency. Figure 2 illustrates the structure of the proposed meta‐particle and the mechanism of the phase and amplitude manipulation. Two boards with the same structure are opposite along the z‐axis with a rotation angle of 90°. The detailed structures of each board are shown in Figure 2b,c, containing a metal patch with a 45° tilt with two varactor diodes in parallel and a metal grid with a gap for the DC bias line. The key dimensions are *t_sub_* = 4 mm, *t_air_* = 3 mm, *p* = 25 mm, *l* = 24.75 mm, *a* = 5 mm, *b* = 8 mm, *c* = 5 mm, *gap* = 0.5 mm, *d* = 6.25 mm, *x_g_* = 1.25 mm, and *w_g_* = 1.25 mm. The varactor diodes (SMV2019‐079LF) on the upper and lower boards are controlled by bias voltages *V_1_* and *V_2_*_,_ respectively. The capacitance of the adopted varactor diodes can vary from 0.3 to 2.22 pF when the control voltage changes from 20 to 0 V [50]. Though the datasheet of the varactor only provides a discrete relationship between capacitance and voltage, the voltage corresponding to any capacitor within the control range can be calculated through spline interpolation. The detailed information of the relationship between capacitance and voltage is given in Note S1. Combined with the SPICE model given in the datasheet, the simulation of the metaparticle under different bias voltages, *V_1_* and *V_2,_* can be conducted. When regulating the proposed metasurface, PWM signals with adjustable duty cycle generated from the FPGA are converted into a DC signal through a low‐pass filter and an analog amplifier, and therefore supply the bias voltages to the proposed metasurface. Details of the voltage control system will be provided in Note S2.

The manipulation principle of the metasurface is illustrated in Figure 2d,e. The metal grids on both sides of the meta‐particle serve as polarization selectors. The x‐polarized incident EM wave will interact with the meta‐particle, while the metal grid will reflect the y‐polarized incident EM wave. The polarized states of x‐polarized incident waves will transfer into y‐polarized after interacting with the x‐shaped patch and the varactors. Since there are two metal grids that rotate 90° on both sides of the meta‐particle, the x‐polarized incident wave undergoes multiple reflections between the two metal grids before transmission, thereby improving the polarization conversion efficiency. To analyze the principle of amplitude and phase manipulation, we first decompose the EM field into the u and v axes, assuming the transmissive efficiency of the u‐polarized and v‐polarized EM wave of the given meta‐particle as *T_uu_* and *T_vv_*.

Since the rotation angle between the *uov* axis and the *xoy* axis is 45°, the relationship between *xy*‐polarized EM wave *E_x_* and *E_y_* and *uv*‐polarized EM wave *E_u_* and *E_v_* can be described as:

(1) $$\left[\begin{array}{c} E_{u}\\ E_{v} \end{array}\right]=Rot\;\left[\begin{array}{c} E_{x}\\ E_{y} \end{array}\right]=\left[\begin{array}{cc} \frac{\sqrt{2}}{2} & \frac{\sqrt{2}}{2}\\ -\frac{\sqrt{2}}{2} & \frac{\sqrt{2}}{2} \end{array}\right]\;\left[\begin{array}{c} E_{x}\\ E_{y} \end{array}\right]$$

where the matrix *Rot* is the rotation matrix. With two polarized selectors on both sides of the meta‐particle, the relationship between incident wave *E_inc_* and transmission wave *E_tran_* can be described as:

(2) $${\left[\begin{array}{c} E_{x}\\ E_{y} \end{array}\right]}_{tran}=\left[\begin{array}{cc} 0 & 0\\ 0 & 1 \end{array}\right]\;Rot^{-1}\left[\begin{array}{cc} T_{uu} & 0\\ 0 & T_{vv} \end{array}\right]Rot\left[\begin{array}{cc} 1 & 0\\ 0 & 0 \end{array}\right]{\left[\begin{array}{c} E_{x}\\ E_{y} \end{array}\right]}_{inc}$$

Therefore, the transmissive and polarization conversion efficiency can be described as *T_xy_* and is defined by the following equation:

(3) $$T_{xy}=\frac{T_{uu}-T_{vv}}{2}\;$$

when we assign *T_ux_ = cos(*45°*)T_uu_* and *T_vx_ = ‐sin(*45°*)T_vv_*, the relationship between *T_xy_*, *T_ux,_* and *T_vx_* can be intuitively given by adding vectors, as Figure 2e shows. By applying different *V_1_* and *V_2_*, the equivalent capacitance of varactor diodes *C*_1_ and *C*_2_ can be changed respectively, resulting in different phase and amplitude of *T_ux_* and *T_vx_*, so that the transmissive parameter *T_xy_* can be manipulated.

The analysis above gives a qualitative analysis of transmissive metasurface design. Since it is hard to obtain a universal formula to describe the EM response of the metal patches in the polarization‐flipping cavity, we conduct numerical simulations through a commercial software, Computer Simulation Technology (CST) Microwave Studio, to obtain the detailed relationship between the capacitance value and the transmission efficiency. The qualitative analysis can be verified through the results of simulations. To illustrate the powerful manipulation ability of the proposed meta‐particle, we select two groups of coding statuses to implement the multi‐bit phase manipulation and amplitude‐phase joint manipulation, respectively.

**FIGURE 3:**
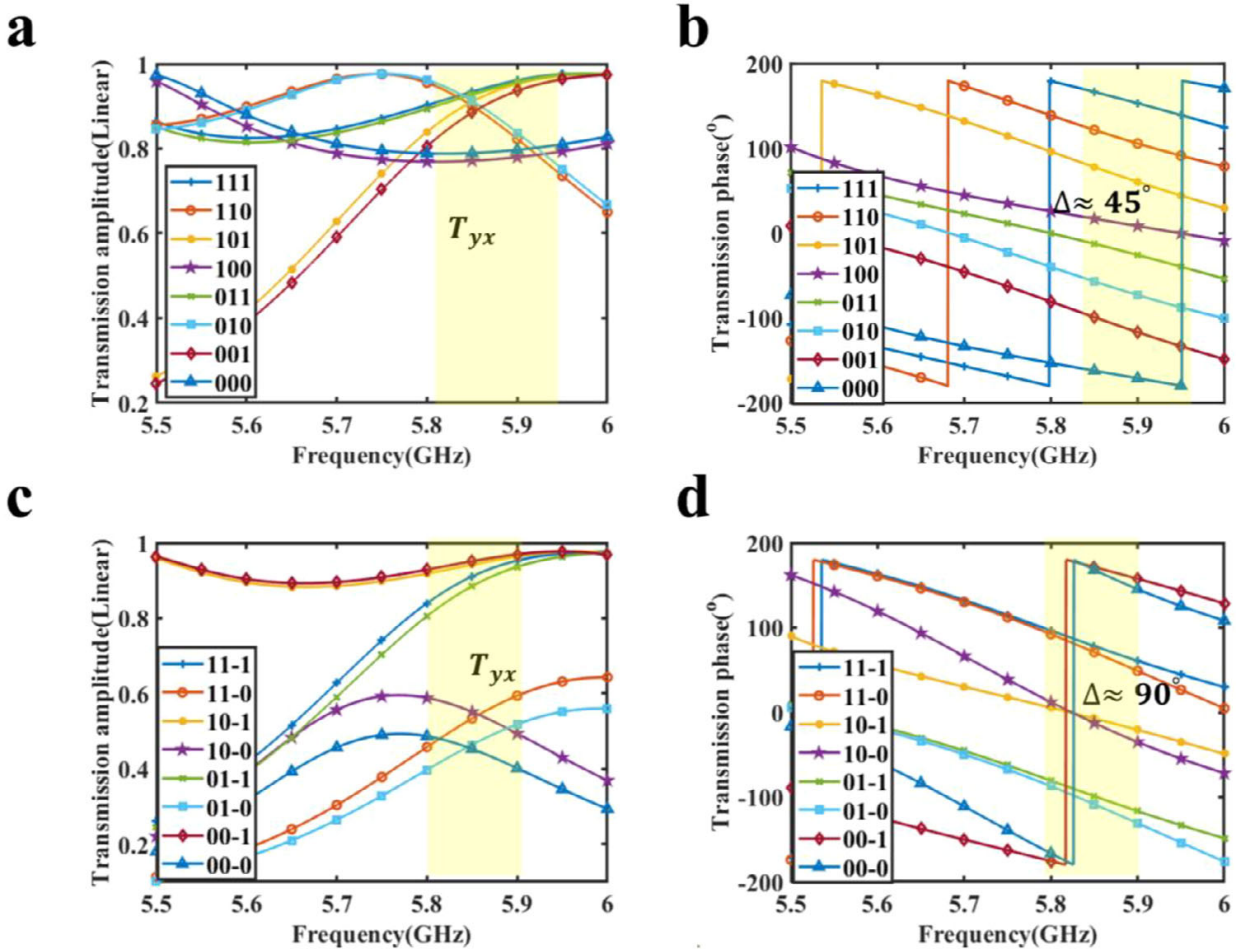
(a,b) The simulation results of the transmission amplitude and phase of the meta‐particles in the 3‐bit phase coding mode. (c,d) The simulation results of the transmission amplitude and phase of the meta‐particles in the phase‐amplitude joint coding mode.

Table 1 shows the coding strategy of the given meta‐particle serving as a 3‐bit phase coding unit, in which the phase response of the meta‐particle is divided into eight states spaced 45° apart, while the amplitude responses of the meta‐particle in 8 coding states are nearly the same. The simulation results of 3‐bit phase manipulation are shown in Figure 3a,b. For convenience, we define the symbol *T*_yx_ to denote the transmission coefficient from x‐polarization to y‐polarization. Figure 3a shows that all the transmissive amplitudes are higher than −3 dB from 5.75 to 5.95 GHz, and the amplitude difference between different coding statuses is smaller than 2 dB from 5.78 to 5.95 GHz. According to Figure 3b, the meta‐particle has eight different transmission phases from 5.7 to 6 GHz, and the phase difference between the two adjacent coding states is 45° at 5.95 GHz. Considering the consistency of the transmission amplitude and phase gradient, we define the operating bandwidth of the metasurface in the 3‐bit coding mode as 5.85–5.95 GHz, where the proposed meta‐particle has a high transmission amplitude and uniform phase encoding state. To directly show the numerical quantitative indicators of the proposed meta‐particle, we list the bandwidth and the stability of transmission amplitude and phase in Table 2. The simulation results show that this meta‐particle can achieve 3‐bit phase manipulation in the sub‐6G band while maintaining a high transmission amplitude, indicating broad application prospects in mobile communication, IoTs, and other scenarios.

**TABLE 1:** The strategy of multi‐bit phase coding mode (3‐bit phase coding).

Code	Phase	*C_1_* (pF)	*V_1_* (V)	*C_2_* (pF)	*V_2_* (V)	Code	Phase	*C_1_* (pF)	*V_1_* (V)	*C_2_* (pF)	*V_2_* (V)
000	0°	0.3	20	0.9	3.44	100	180°	0.9	20	0.3	20
001	45°	0.3	20	0.5	6.62	101	225°	0.5	6.62	0.3	20
010	90°	1.5	1.02	0.5	6.62	110	270°	0.5	6.62	1.5	1.02
011	135°	1.2	2	0.4	9	111	315°	0.4	9	1.2	2

**TABLE 2:** The performance of the proposed meta‐particle in 3‐bit phase coding mode.

Bandwidth (GHz)	Maximum *T_xy_* (dB)	Minimum *T_xy_* (dB)	Maximum *Δφ*	Minimum *Δφ*
5.85–5.95	−0.52	−2.4	55**°**	36**°**

*Δφ: phase difference between the two adjacent coding states.

Table 3 shows the coding strategy of the given meta‐particle serving as a phase‐amplitude joint manipulation unit, where the phase response of the metasurface is divided into four states spaced 90° apart, while the amplitude response of the metasurface is divided into 0.5 and 1. All these responses can be manipulated independently by endowing different capacity values. For simplicity, we represent the status of the proposed meta‐particle with a three‐digit binary code, in which the last bit represents the amplitude code and the top two bits represent the phase code. For example, the code of the metasurface can be represented as 00–1 when the phase code is 00, and the amplitude code is 1.

**TABLE 3:** The strategy of phase‐amplitude joint coding mode (1‐bit amplitude and 2‐bit phase).

Phase Code	Amp Code	Code	*C*_1_ (pF)	*V_1_* (V)	*C*_2_ (pF)	*V_2_* (V)	Phase Code	Amp Code	Code	*C*_1_ (pF)	*V_1_* (V)	*C*_2_ (pF)	*V_2_* (V)
00	0	00‐0	0.4	9	0.475	7.11	00	1	00‐1	0.3	20	0.5	6.62
01	0	01‐0	0.6	5.51	0.5	6.62	01	1	01‐1	1	2.89	0.4	9
10	0	10‐0	0.475	7.11	0.4	9	10	1	10‐0	0.5	6.62	0.3	20
11	0	11‐0	0.5	6.62	0.6	5.51	11	1	11‐1	0.4	9	1	2.89

*Amp Code = 0, 1: Amplitude = 0.5, 1; Phase Code = 00, 01, 10, 11: Phase = 0°, 90°, 180°, 270°.

Figure 3c,d shows the simulation results of the meta‐particle under the phase‐amplitude joint coding strategy. The transmission amplitude of the meta‐particle is shown in Figure 3c, when the amplitude coding state is 1, the transmission amplitude of the meta‐particle ranges from 0.8 to 0.97 (−2 to −0.5 dB) from 5.8 to 5.9 GHz, and when the amplitude coding state is 0, the transmission amplitude of the meta‐particle ranges from 0.4 to 0.6 (−6.5 to −5 dB) from 5.8 to 5.9 GHz, expressing that the meta‐particle has good amplitude consistency in amplitude coding. The transmission phase is shown in Figure 3d. The difference in transmission phase between different phase coding states is around 90°, while the phase difference within the same phase coding state is less than 5° in the range of 5.8 – 5.9 GHz. Considering the consistency of the transmission amplitude and phase gradient, we define the operating bandwidth of the metasurface in the phase‐amplitude joint coding mode as 5.8–5.9 GHz. To directly show the numerical quantitative indicators of the proposed meta‐particle, we list the bandwidth and the stability of transmission amplitude and phase in Table 4. The simulation results show that the metasurface can independently manipulate phase and amplitude using the given coding strategy, demonstrating its excellent capability for programmable joint phase‐amplitude manipulation and broad application prospects in the sub‐6G band.

**TABLE 4:** The performance of the proposed meta‐particle in phase‐amplitude joint coding mode.

Bandwidth (GHz)	*T_xy_* with Amp Code = 0 (linear)	*T_xy_* with Amp Code = 1 (linear)	Maximum *Δφ_0_*	Maximum *Δφ_1_*	Minimum *Δφ_1_*
5.8–5.9	0.41–0.57	0.82–0.95	14°	100°	86°

*Δφ0: phase difference between the two different Amp Code with the same Phase Code

*Δφ1: phase difference between the two adjacent phase coding states.

### 2.2 Simulation of the Metasurface

To validate the function of the metasurface, we simulate the performance of a metasurface array consisting of 16 × 16 meta‐particles in three typical applications with different coding modes, including orbital angular momentum (OAM) beam generation, hologram imaging, and multi‐beam forming.

When working in multi‐bit phase coding mode, this metasurface can be used as an OAM wave generator. To avoid the petal‐like amplitude structure in 1‐bit OAM generating and reduce the difficulty of measurement, we use phase modulation to simultaneously realize OAM wave generation and focusing. Therefore, the phase distribution on the metasurface can be described as

(4) $$\varphi \;\left(x,y\right)=l\tan^{-1}\frac{x-x_{0}}{y-y_{0}}+\frac{2\pi }{\lambda }\sqrt{{\left(x-x_{1}\right)}^{2}+{\left(y-y_{1}\right)}^{2}+{z_{1}}^{2}}$$

where *x_0_* and *y_0_* are the coordinates of the center of the metasurface, *l* is the order of the OAM wave determining the direction and period of phase rotation, and *x_1_*, *y_1_*, and *z_1_* are the coordinates of the center of the focal point of the OAM wave. In this paper, we simulated the performance of generating OAM waves with *l* = 2 in the plane *z_1_* = 200 mm through different phase coding strategies, including 1‐bit, 2‐bit, and 3‐bit phase coding strategies. When adopting the N‐bit phase coding strategy, by rounding down, the phase you learn can be quantified into 2^N^ codes, and the coding state of a meta‐particle can be determined by:

(5) $$code\;\left(i,j\right)=\lfloor \frac{\varphi \left(x_{i},y_{j}\right)}{2\pi }2^{N}\rfloor \;$$

where (*x_i_, y_i_*) is the coordinate of the center of a given meta‐particle, and *φ* ranges from 0 to 2π.

**FIGURE 4:**
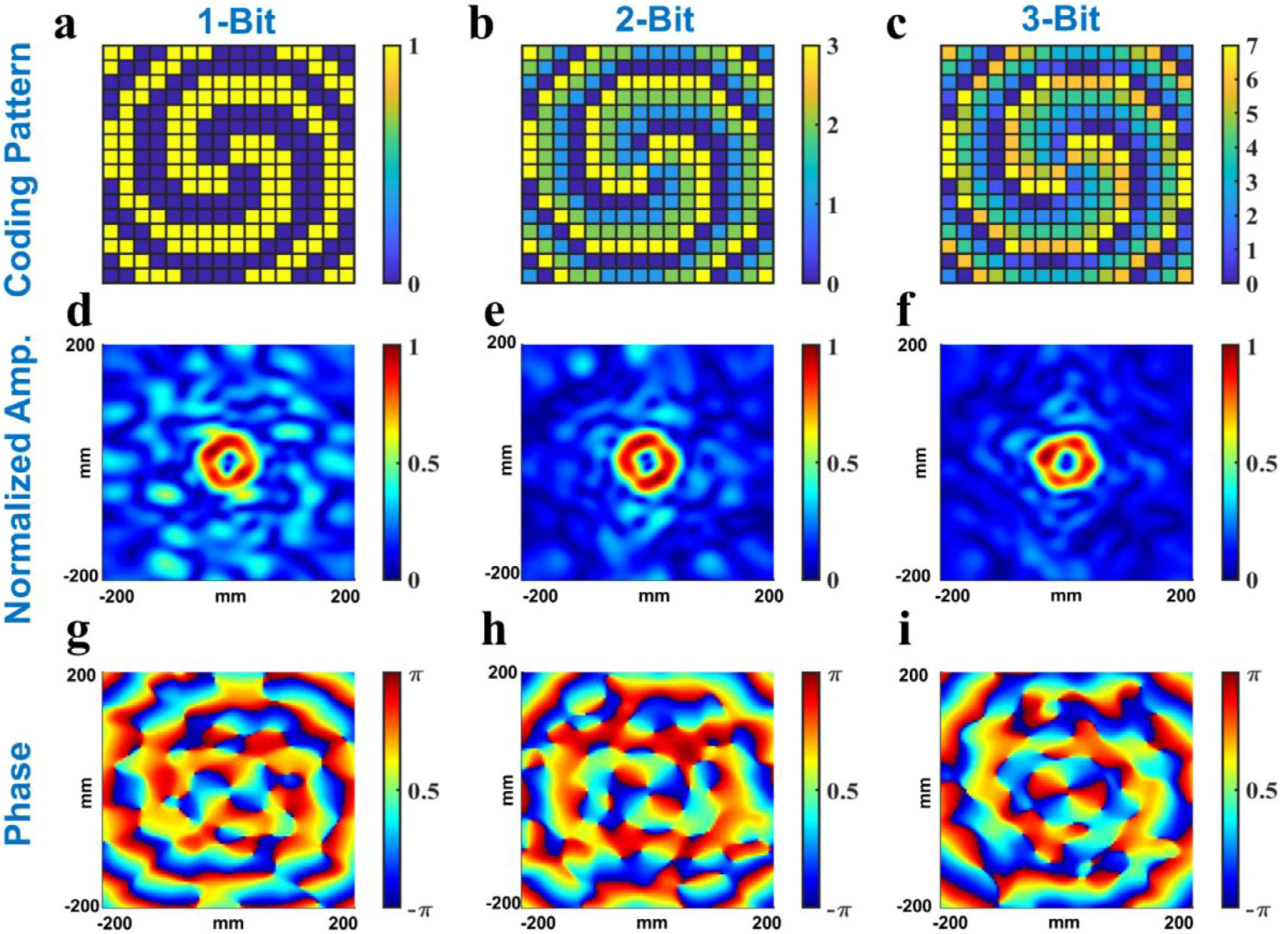
(a–c) The coding patterns of the metasurface when adopting 1‐bit, 2‐bit, and 3‐bit phase coding strategies. (d–f) The normalized amplitude of the OAM wave in the plane *z* = 200 mm when adopting 1‐bit, 2‐bit, and 3‐bit phase coding strategies. (g–i) The phase distribution of the OAM wave in the plane *z* = 200 mm when adopting 1‐bit, 2‐bit, and 3‐bit phase coding strategies.

Figure 4 shows the simulation results of OAM wave generations at 5.9 GHz through different coding strategies. Figure 4a–c shows the coding patterns of the proposed metasurface serving as an OAM wave generator with different coding strategies. The OAM wave amplitude distributions in the plane *z* = 200 mm in full‐wave simulation are shown in Figure 4d–f, indicating that the energy of the transmitted wave becomes more concentrated as the number of encoded bits increases. Besides, Figure 4g,h show the phase distribution of OAM wave generation at the same plane, where it becomes more uniform at the focal ring with increasing the number of encoded bits. Hence, this metasurface can provide better performance due to its multi‐bit phase control ability and maintain the same flexibility compared with the previous research based on 1‐bit phase programmable metasurfaces.

**FIGURE 5:**
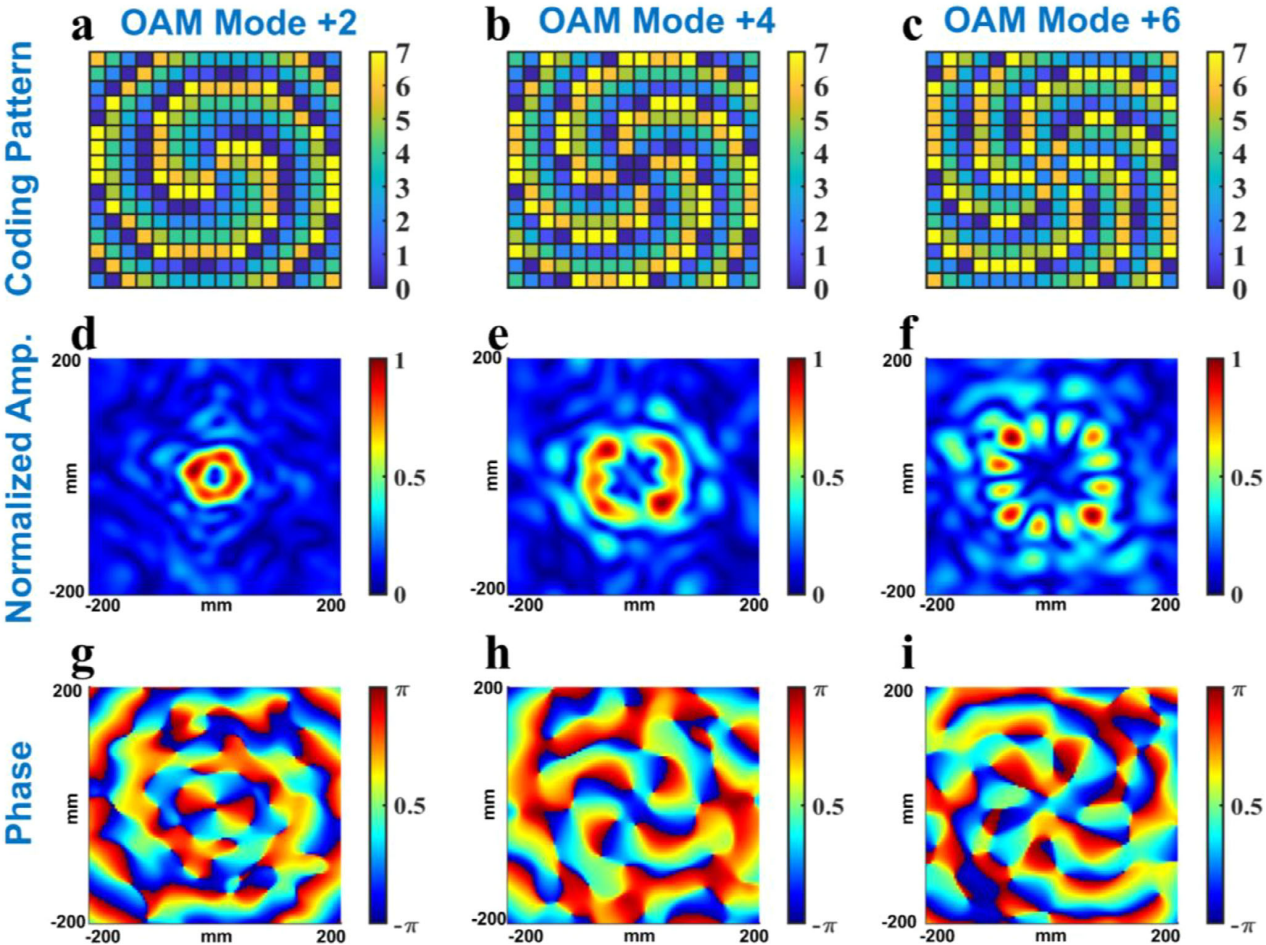
(a–c) The coding patterns of the metasurface when serving as an OAM wave generator with *l* = +2, +4, and +6. (d–f) The normalized amplitude of the OAM wave in the plane *z* = 200 mm with *l* = +2, +4, and +6. (g–i) The phase distribution of the OAM wave in the plane *z* = 200 mm with *l* = +2, +4, and +6.

Figure 5 shows the coding patterns and simulation results of the proposed metasurface generating OAM waves with *l* = +2, +4, and +6, illustrating the dynamic OAM wave generation capability of the proposed metasurface. Compared with previous studies based on a fixed metasurface that can approximately provide continuous phase modulation, this metasurface can dynamically change its function without sacrificing too much regulatory performance due to the programmable feature. The simulation result agrees with the theoretical analysis, indicating the advantage of the multi‐bit phase coding strategy and the potential application in OAM antennas. To further estimate the quality of OAM beam generation, we conduct numerical analysis on both the focusing efficiency and mode purity, and the analysis results are shown in Table 5. The numerical analysis reveals that with an increase in coding bits, the focusing efficiency and mode purity also increase. Conversely, with a given coding strategy and a limited aperture, the OAM performance deteriorates with an increase in OAM level.

**TABLE 5:** Numerical analysis on OAM generation.

Coding Strategies	OAM Level	Mode Purity	Focusing efficiency
1‐bit	2	90.7%	37.8%
2‐bit	2	92.8%	59.2%
3‐bit	2	95.2%	69.5%
3‐bit	4	84.8%	55.6%
3‐bit	6	67.5%	47.8%

Another near‐field application is holography imaging. For a transmissive metasurface, the transmissive efficiency and ability to precisely control phase directly decide the imaging quality in holographic image systems. Hence, with high transmissive efficiency and precise multi‐bit phase manipulation ability, the proposed metasurface can be of great potential application in holographic imaging.

To get the coding pattern of the metasurface, we adopt an imaging algorithm based on the Gerchberg–Saxton (GS) algorithm. The conventional GS algorithm uses Fast Fourier Transform (FFT) and Inverse Fast Fourier Transform (IFFT) to modify optical diffraction and inverse diffraction. However, compared with optical systems, a microwave system has a smaller aperture and a shorter imaging distance. Therefore, we modify the GS algorithm with Huygens' Principle and Kirchhoff's diffraction theory. By treating each point on the metasurface as a sub‐wave source, the relationship between the EM field in the imaging plane *G(x,y)* and the field on the metasurface *F(x,y)* can be described as:

(6) $$G\;\left(x_{0},y_{0}\right)=\frac{1}{i\lambda }\;\int \;\;\;\int \frac{F\left(x,y\right)e^{-i\frac{2\pi }{\lambda }\sqrt{{\left(x-x_{0}\right)}^{2}+{\left(x-x_{0}\right)}^{2}+z^{2}}}}{\sqrt{{\left(x-x_{0}\right)}^{2}+{\left(x-x_{0}\right)}^{2}+z^{2}}}dxdy$$

where *λ* is the wavelength of the EM wave, *(x,y)* is the coordinate on the metasurface, *(x_0_, y_0_)* is the coordinate on the imaging plane, and *z* is the distance between the metasurface and the imaging plane. After finishing the calculation of the phase distribution of the metasurface, we can obtain the codes of the metasurface through Equation (5). In this section, we simulate the holography imaging system through CST at 5.85 GHz. The scale of the metasurface is 16 × 16. The distance between the metasurface and the hologram is 400 mm.

**FIGURE 6:**
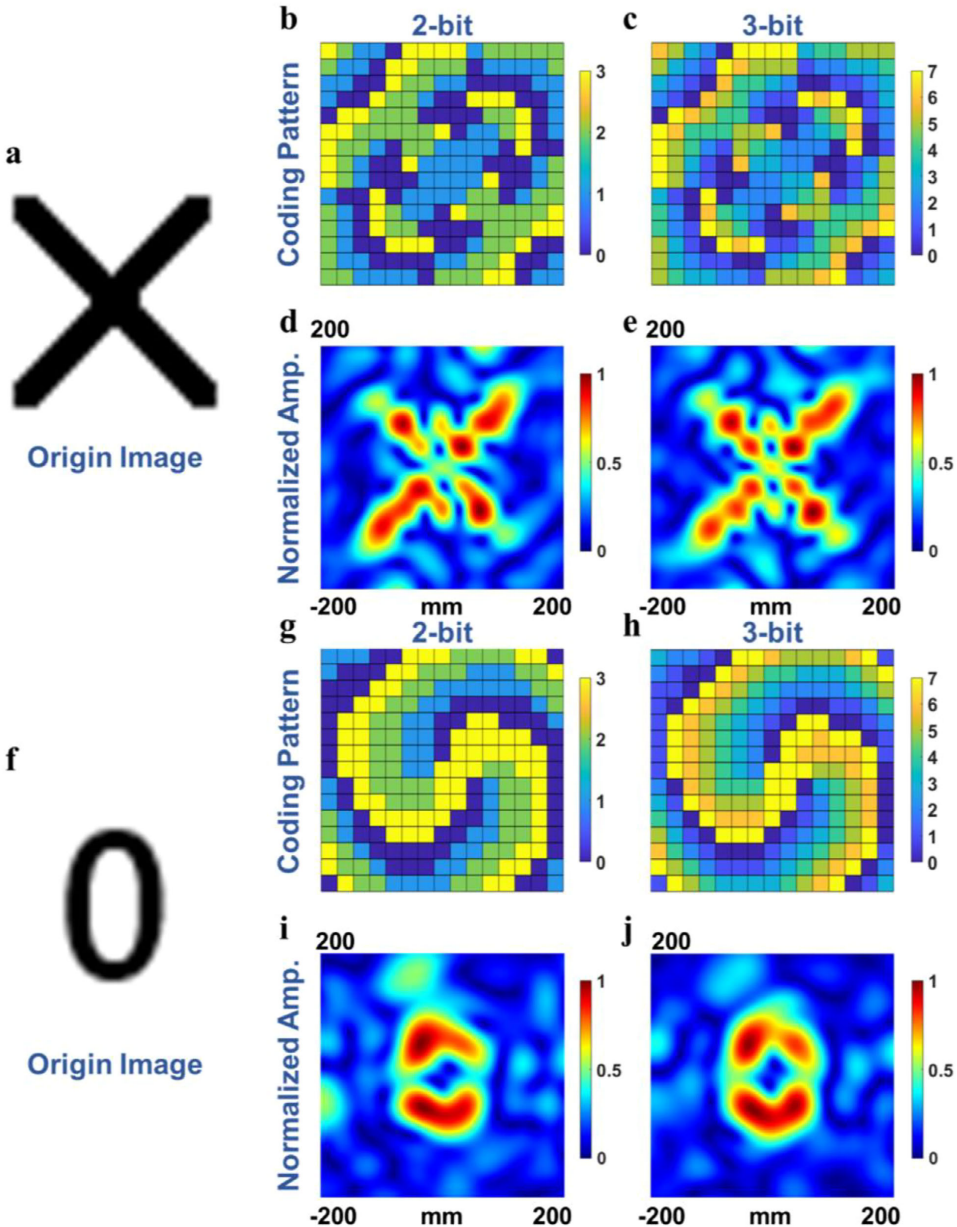
(a) origin image of character “X”. (b–e) the coding patterns and amplitude distributions of the character “X” via the 2‐bit and 3‐bit phase coding strategy. (f) original image of character “0”. (g–j) the coding patterns and amplitude distributions of the character “0” via the 2‐bit and 3‐bit phase coding strategy.

The simulated results of “X” and “0” holography imaging are illustrated in Figure 6. Figure 6b,c,g, and h show the coding patterns on the metasurface when realizing “X” and “0” holograms. The imaging results are shown in Figure 6d,e,i,j. Compared with the 2‐bit phase coding strategy, the 3‐bit one can realize more precise phase control by increasing the number of codes, and the imaging results show the holographic image quality under different coding strategies, indicating that by increasing the number of codes, the quality of imaging can be improved. The simulation results show that the proposed metasurface uses only an 8*λ* × 8*λ* aperture to achieve a similar imaging quality to the previous research [4] of more than 10*λ* × 10*λ* aperture, implying the powerful ability and potential application of the proposed metasurface in holography imaging. Though certain deviations between the imaging result and the original image occur in the simulations, considering the aperture of the metasurface, the quality of the imaging results is acceptable.

Apart from the near‐field manipulation, far‐field manipulation is another important function of a metasurface. To illustrate the phase‐amplitude joint manipulating capacity of the proposed metasurface, multi‐beam forming is achieved in the far field. As is known, the generalized Snell's law is usually adopted in traditional metasurface beamforming. For a reflective metasurface with a normally incident plane EM wave, the phase gradient *k_φ_* on the metasurface can be calculated by Equation 7 [1] if the reflection direction *θ_r_* is given.

(7) $$k_{\varphi }=\frac{2\pi }{\lambda }\;\sin\theta_{r}$$

**FIGURE 7:**
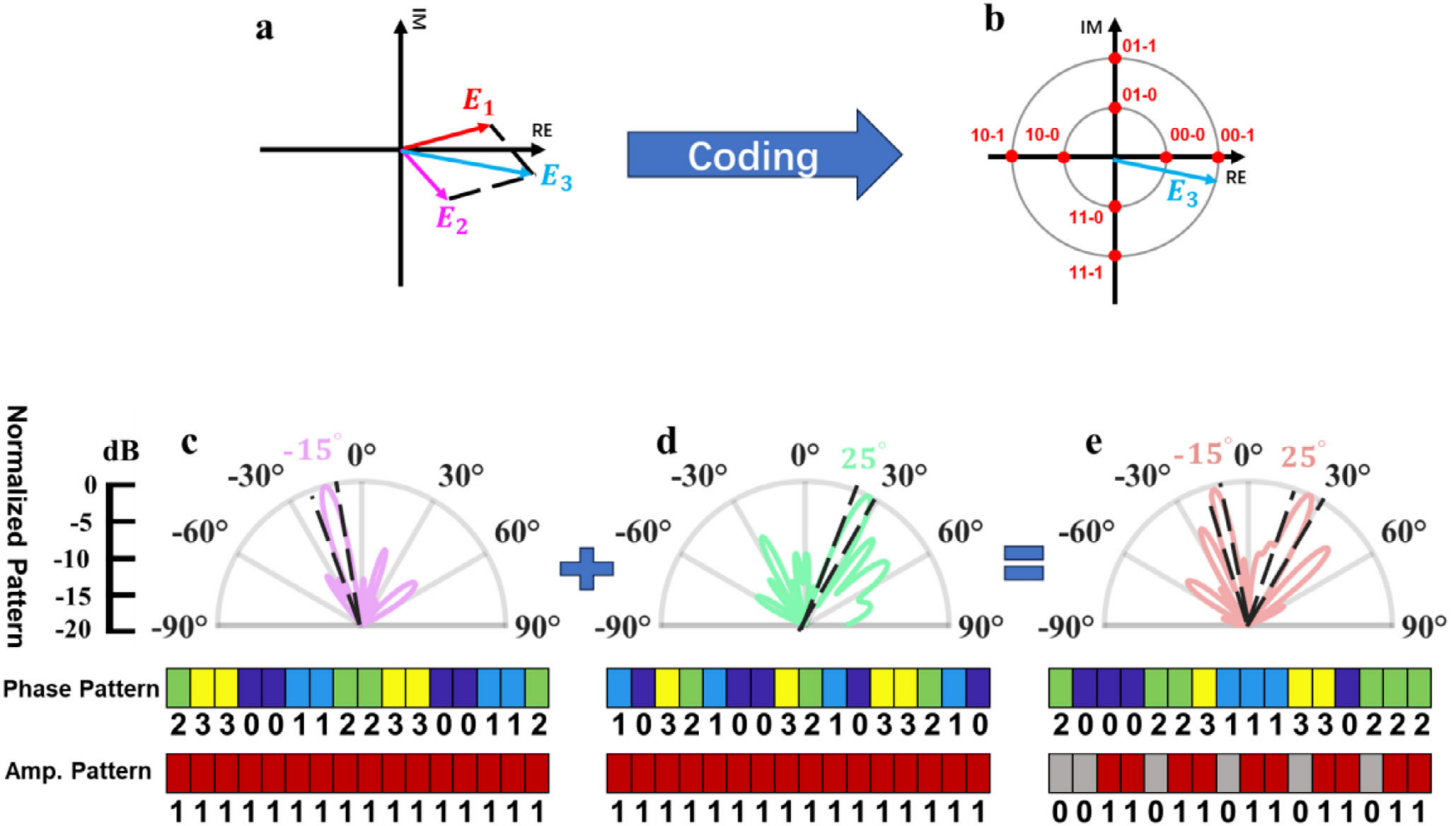
(a) Schematic of the addition of amplitude and phase response. (b)Schematic of amplitude‐phase joint coding strategy. (c–e) The simulation results and phase and amplitude coding patterns of adding two lobes at −15° and 25° using the proposed metasurface with a 1‐bit amplitude and 2‐bit phase coding ability.

With the phase gradient *k_φ_*, the phase distribution *φ(x,y)* can be calculated. However, in the realization of multi‐beam forming, this theory is not applicable. One possible solution to multi‐beam forming is array synthesis based on the Fourier transform, with the disadvantage of increasing calculation complexity. In 2018, the addition theorem [38] for coding and digital metasurface was proposed to achieve multi‐beam forming by using complex coding states, but there would still be some deterioration in the outcome due to the balance of phase and amplitude manipulation. Here, we propose a method to fulfill multi‐beam forming by extending the metasurface‐based addition operation by adding amplitude control in a programmable metasurface. Figure 7a,b directly show the addition and coding operation of the programmable metasurface. For a meta‐particle with continuous amplitude and phase response, the characteristic of a metasurface can be represented by a vector in the complex plane, and when adding two different meta‐particles *E_1_* and *E_2_*, the result *E_3_* can be determined by the parallelogram rule. In the proposed programmable metasurface, the phase and amplitude response are discretized, and we can code a meta‐particle according to the nearest neighbor rule, as shown in Figure 7b.

To illustrate the principle of the addition operation of metasurfaces, we assume that the amplitude‐phase distributions of a metasurface in two different functions are *F_1_(x,y)* and *F_2_(x,y)*. If we need a metasurface to fulfill the two functions simultaneously, according to the linear model in EM wave propagation, the amplitude‐phase distribution of a metasurface is

(8) $$F_{3}\;\left(x,y\right)=F_{1}\;\left(x,y\right)+F_{2}\left(x,y\right)$$

Through the amplitude‐phase distribution of the metasurface, the code of the meta‐particle can be determined by the phase and amplitude of the geometric center of the meta‐particle and the coding method.

Here, we use a multi‐beam forming as an example to show how the addition operation works. First, we design a phase distribution on the metasurface with a beam at −25° and 15°, respectively, and then we add these two distributions to obtain a phase‐amplitude joint distribution of the metasurface to form beams at −15° and 25° simultaneously. Figure 7c–e show the far‐field patterns and coding patterns when the metasurface forms a main lobe at −15° and 25°, and simultaneously forms main lobes at −15° and 25° with a phase‐amplitude coding strategy, respectively. Because the beams occur in the yoz plane, the 2D coding patterns are degenerated to a 1D coding sequence in columns. For single beam forming, amplitude values of all the elements are equal to 1, indicating the uniform amplitude distribution and making full use of the transmission EM waves. When adding two different phase distributions, the amplitude distribution of the additional result is no longer uniform. Hence, the metasurface can simulate the scenario of linear addition in the EM field with uneven amplitude distribution. The simulation results show that a metasurface can fulfill the adding operation even with limited phase‐amplitude joint coding ability. Thus, the ability of amplitude‐phase joint coding and programmability can contribute to developing multifunctional metasurfaces and reduce the design complexity.

## 3 Experimental Results

To validate the performance of the metasurface, we fabricate a 250mm × 250 mm prototype using the printed circuit board (PCB) technology with 8 × 8 meta‐particles. 8 × 8 × 4 varactor diodes are employed to control the transmitted patterns of the metasurface. It should be noticed that the fabricated sample is smaller than that in simulations for the purpose of simplifying processes and reducing costs. Though the reduction in the array scale can result in a decline in focusing efficiency, imaging resolution, and OAM mode purity in near‐field experiments, and cause broadening of the main lobe and deterioration of gain in far‐field beamforming, the measured results can still verify the proposed design, and the numerical analysis of the difference between an 8 × 8 array and a 16 × 16 array will be discuss in the Supporting Information.

**FIGURE 8:**
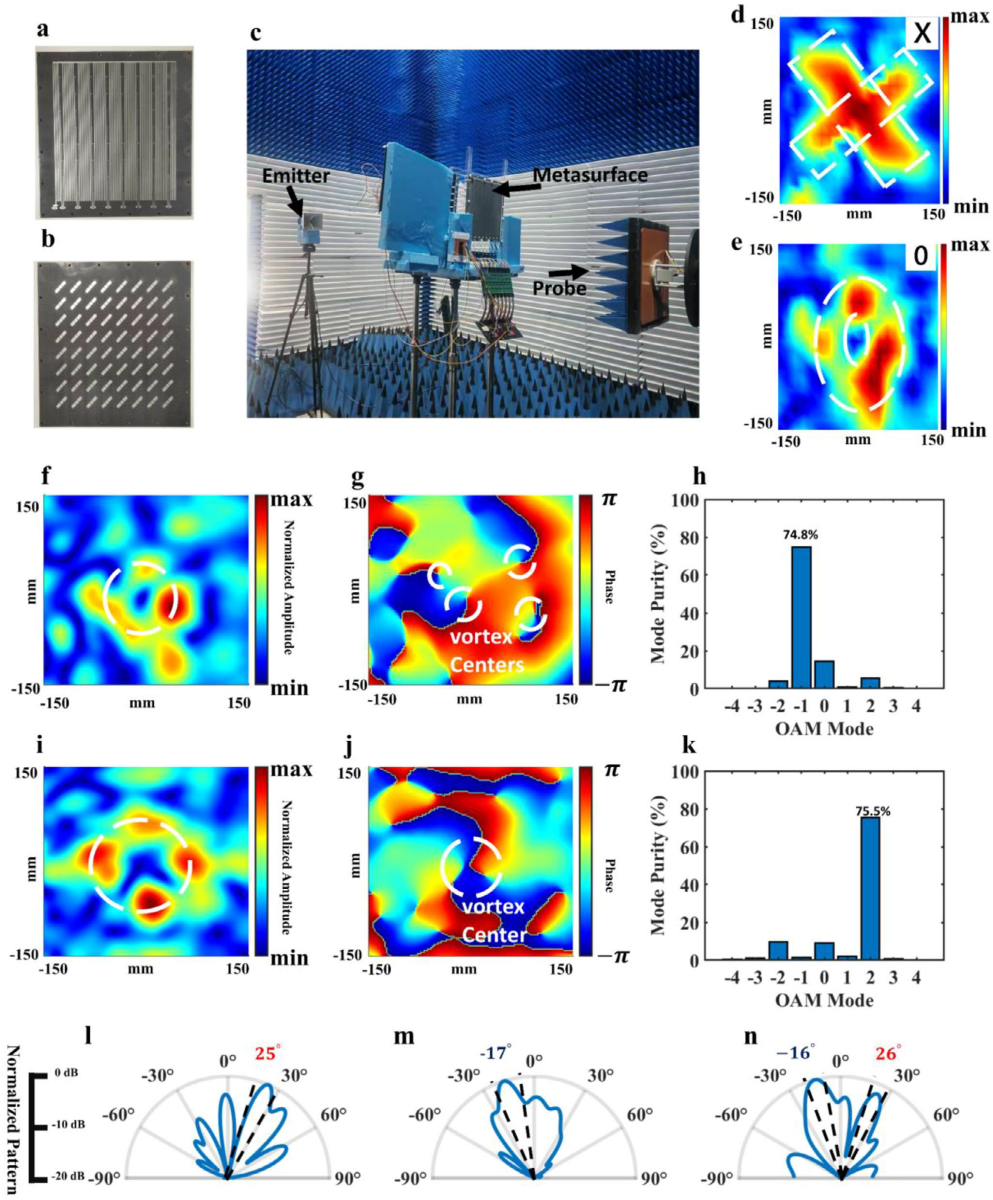
(a,b) The structure of the proposed metasurface with 8 × 8 meta‐particles. (c)The test environment of the near‐field microwave anechoic chamber. (d,e) The original graphs and the measurement results of the holographic imaging of ‘X’ and ‘0’. (f–h) The amplitude distribution, phase distribution, and OAM mode distribution of the OAM wave when *l* = −1. (i–k) The amplitude distribution, phase distribution, and OAM mode distribution of the OAM wave when *l* = 2. (l–n) The beam forming results when the main lobe is pointing at 25° and −15°, and dual‐beam performance.

The prototype is measured in a near‐field microwave anechoic chamber to verify the function of the proposed metasurface. Figure 8a–c shows the details structure and the experimental environment. The horn antenna is placed 2m from the metasurface to send the planar incident wave. We conduct holographic imaging and OAM wave generation to verify the phase manipulation capability, and conduct beamforming and verify the addition operation by phase‐amplitude joint manipulation.

When conducting the holographic imaging, the distance between the metasurface and the imaging plane is 200 mm, and the imaging results are measured at 5.95 GHz. The imaging results of this metasurface under different codes can be obtained by scanning the electric field of the imaging plane. In the experiment, we take ‘X’ and ‘0’ as imaging examples; the original graphs and imaging results are shown in Figure 8d,e. The imaging result of ‘X’ is in good agreement with the original shape, while the imaging result of ‘0’ has a distortion compared with the original image. Although the amplitude distribution of the imaging result of ‘0’ is uneven, the original image can still be identified. The imaging results show that the proposed metasurface can fulfill a satisfactory imaging effect even in a small aperture, indicating its broad application prospects in holographic imaging and electromagnetic field information modulation.

The measurement setup is similar when conducting OAM beams generation, where the focusing plane is also 200 mm. Taking *l* = −1 and *l* = 2 as examples, the measurement results of OAM beams are shown in Figure 8f–k. The amplitude distribution of the OAM on the focusing plane shows certain focusing characteristics, and the phase distribution shows certain vortex characteristics. The measurement results show that there are four vortex centers when *l* = −1, and only one vortex center when *l* = 2, which is consistent with the simulation and analysis in the previous text and the Supporting Information. When *l* = −1, the focusing efficiency is 51.5%, and when *l* = 2, the focusing efficiency is 57.2%. Furthermore, we select the electric field within the focused annular area (as shown by the dotted circular ring in Figure 8f,i to calculate the purity of the OAM mode, and the calculated results are shown in Figure 8h,k. The OAM mode's purity reaches 74.8% and 75.5% in *l* = −1 and *l* = 2, respectively. The experimental results of OAM beam generation are in good agreement with the design objective and theoretical analysis, indicating the potential applications in designing novel antennas and OAM beam multiplexing.

The experiment results of holographic imaging and OAM beam generation are not as satisfactory as the simulation results, which is due to the limited metasurface aperture (3.97λ × 3.97λ), the difference between the varactor diode and its theoretical model, and the scattering interference caused by the feeding cables, microcontroller, and voltage control circuit. One possible way to optimize the performance of the metasurface in nearfield manipulation, including holography imaging and OAM beamforming, is to enlarge the metasurface array and reduce the volume of the control circuit.

To verify the capability of phase‐amplitude joint manipulation of the metasurface, we conduct dual‐beam forming and realize the addition operation at 5.85 GHz. The far‐field scattering characteristics of metasurfaces can be obtained by transforming the measured electric‐field results in a near‐field anechoic chamber. To make sure the measurement position meets the near‐field conditions, the probe is set 300 mm from the metasurface. The measured results are shown in Figure 8l–n. When conducting single beamforming, the positions of the mainlobe are set at −15° and 25°, and the metasurface is coded with a fixed amplitude (the amplitude codes of the meta‐particles are set to “1”) and a 2‐bit phase coding strategy, while the metasurface is coded with a 2‐bit phase and 1‐bit amplitude coding strategy when verifying the addition operation. When the designed main‐lobe points at 25°, the measured result shows that the main‐lobe is at 26° and the side‐lobe level is −4 dB. When the designed main‐lobe points at −15°, the measured result shows that the main‐lobe is at −17° and the side‐lobe level is −4.5 dB. Due to the limited metasurface aperture and errors caused by phase quantization, the main beam is broadened, and the side lobe level is increased. The measured result of the addition operation is shown in Figure 8m, and the two lobes exist at −16° and 26°, which is in good agreement with the design objective. The deviation of the beam position might be caused by measurement errors and the difference between the varactor diode and its theoretical model. The measured results of the proposed metasurface show a powerful ability in far‐field spatial electromagnetic wave modulation, indicating the potential applications in remote sensing and wireless communication.

## 4 Conclusions

We propose a novel multi‐functional programmable transmissive metasurface providing both phase programmable and phase‐amplitude joint programmable capability. To ensure this property, a meta‐particle with high transmissive efficiency is designed based on the polarization conversion principle. By switching different bias voltages of the embedded varactor diodes, the cross‐polarized transmitted phase and amplitude responses can be uniquely manipulated in real time, showing 3‐bit coding states in multi‐bit phase programmable mode and 1‐bit amplitude and 2‐bit phase coding states in phase‐amplitude joint programmable mode. We conduct simulation and experimental measurements to verify the function of the proposed metasurface. Holographic imaging, OAM beams generation, and beamforming are realized by an 8 × 8 metasurface array in the experiment. To show the advantage of the amplitude‐phase joint manipulation, we also demonstrate the implementation of multi‐beam forming through the addition operation. Good agreements between simulation, measurement, and theoretical analysis can validate the powerful multi‐functional programmable capability of the proposed novel design. Enjoying the profits of simple structure, high transmissive efficiency, precise phase manipulation ability, and amplitude‐phase joint manipulation ability, the proposed metasurface exhibits more outstanding EM control capabilities and broader application prospects compared with previous studies. It can also find great potential in handling different complex tasks due to the feature that the working mode can be adjusted according to the application scenario. We believe that the proposed multifunctional programmable metasurface can be widely utilized in wireless communication, remote sensing, and all fields that require precise EM manipulations.

## Conflicts of Interest

The authors declare no conflict of interest.

## Supporting information




**Supporting File**: advs73487‐sup‐0001‐SuppMat.docx.

## Data Availability

The data that support the findings of this study are available from the corresponding author upon reasonable request.
