# Supplementary material for: Multifunctional Programmable Transmissive Metasurface with Phase and Amplitude Manipulation Capability
Source: Adv Sci (Weinh). 2025 Dec 22;13(13):e18176. doi: 10.1002/advs.202518176 (PMC12955997; doi:10.1002/advs.202518176)
Supplement: Supplementary file 1 — Supporting File: advs73487‐sup‐0001‐SuppMat.docx. [file ADVS-13-e18176-s001.docx]

Supporting Information

Multifunctional Programmable Transmissive Metasurface with Phase and Amplitude Manipulation Capability

Hao Tian Shi^†^, Rui Yuan Wu^†*^, Shi He, Xue Yun Guo, Xiao Qing Chen, Yi Ning Zheng, Bing Bing Zhu, Lei Zhang and Tie Jun Cui^*^

This Supplementary Information includes:

Supplementary Note 1. Method of the manipulation of the varactor diode (SMV2019-079lf).

Supplementary Note 2. The external control circuit that generated DC bias voltages.

Supplementary Note 3. Comparison of odd-mode OAM and even-mode OAM generation based on a 16×16 array.

Supplementary Note 4. Comparison of the performance difference in OAM generation between a 16×16 array and an 8×8 array.

Supplementary References

[1] Skyworks Solutions, SMV2019 To SMV2023 Series: Hyperabrupt Junction Tuning Varactors https://www.skyworksinc.com/-/media/SkyWorks/Documents/Products/201-300/SMV2019_to_SMV2023_Series_200074S.pdf

**Supplementary Note 1. Method of the manipulation of the varactor diode (SMV2019-079lf)**

The SPICE model of the varactor diodes (SMV2019-040lf) is given in the datasheet of Skyworks ^[1]^. Hence, the adopted equivalent circuit in full-wave simulation can be obtained through this SPICE model. Since the varactor diodes in the proposed metasurface are operated in a reverse-biased state, according to the SPICE model in **Supplementary Fig. 1a**, the varactor diodes can be modeled as an RLC series circuit in the CST simulation. The parameters of the equivalent circuit shown in Supplementary Fig. 1b are *R*=4.8Ω, *L*=0.7nH, and *C* depends on the DC bias voltage. To obtain a continuous voltage-capacitance relationship, we performed cubic spline interpolation on the discrete data given in the data sheet, and the interpolation results are shown in Supplementary Fig. 1b.


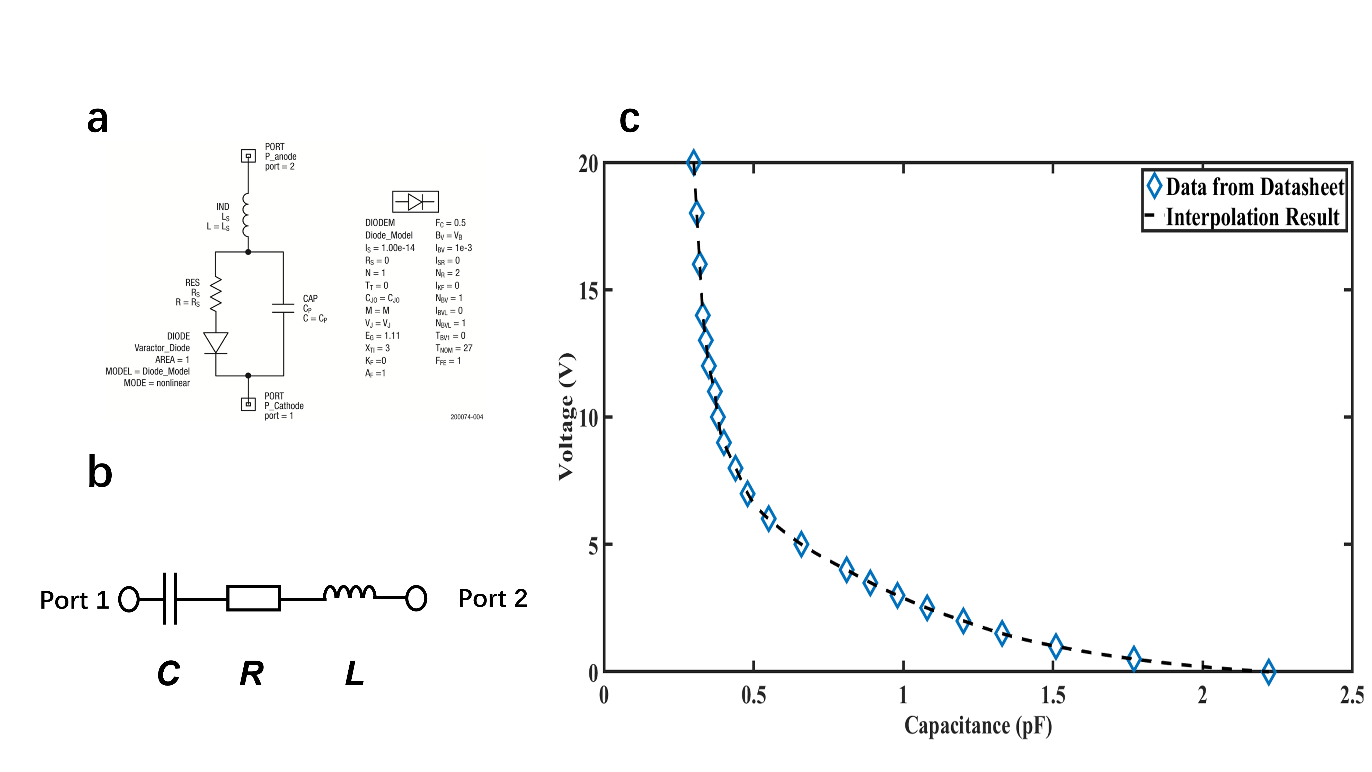


**Supplementary Fig. 1.** The equivalent model of the varactor diode. **a.** the SPICE model of SMV2019 given by the datasheet ^[1]^. **b.** The equivalent circuit adopted in CST simulations. **c.** The relationship between the capacitance and the control voltage, including the data given by Skyworks and the cubic spline interpolation result.

**Supplementary Note 2. The external control circuit that generated DC bias voltages.**

The proposed metasurface is controlled by an FPGA (Cyclone II EP2C5T144C8N). However, the FPGA can only output digital signals with only high-level voltage (3.3V) and low-level voltage(0V). To output a voltage signal that can continuously vary from 0 to 20V and make full use of the Pin resources of the FPGA, we adopt a 50KHz PWM signal to control the varactor. The conversion circuit based on the LM358 Operational amplifier that transfers the PWM signal to a DC bias voltage is shown in **Supplementary Fig. 2a**. When the conversion circuit is working, the PWM signal first goes through a low-pass filter that converts to a DC voltage ranging from 0 to 3.3V, and then goes through an in-phase amplifier that amplifies the voltage to 0-20V. The output DC voltage *V* is defined by the following formula:

$V=dV_{1}\left( 1+\frac{R_{4}}{R_{2}} \right)$ (1)

where *d* is the duty cycle of the PWM signal, and *V_1_* is the value of the high-level voltage of the PWM signal. The values of R2 and R4 in Supplementary Fig. 2a are *R_2_*=10KΩ and *R_4_*=51KΩ. To further demonstrate the performance of the external circuit that generates the control DC voltage, we conduct simulations and measurements on the proposed circuit, and the corresponding results are shown in Supplementary Fig. 2b-2d. The results of the output voltage when the duty cycle is 50% show that it takes about 20ms for the output voltage to reach a steady state, and the ripple of the output voltage is less than 20mV. The measurement results of the output voltages under different duty cycles match the theoretical value well. Despite the measurement error, the design circuit can satisfy our requirement for generating control voltages for the varactor.


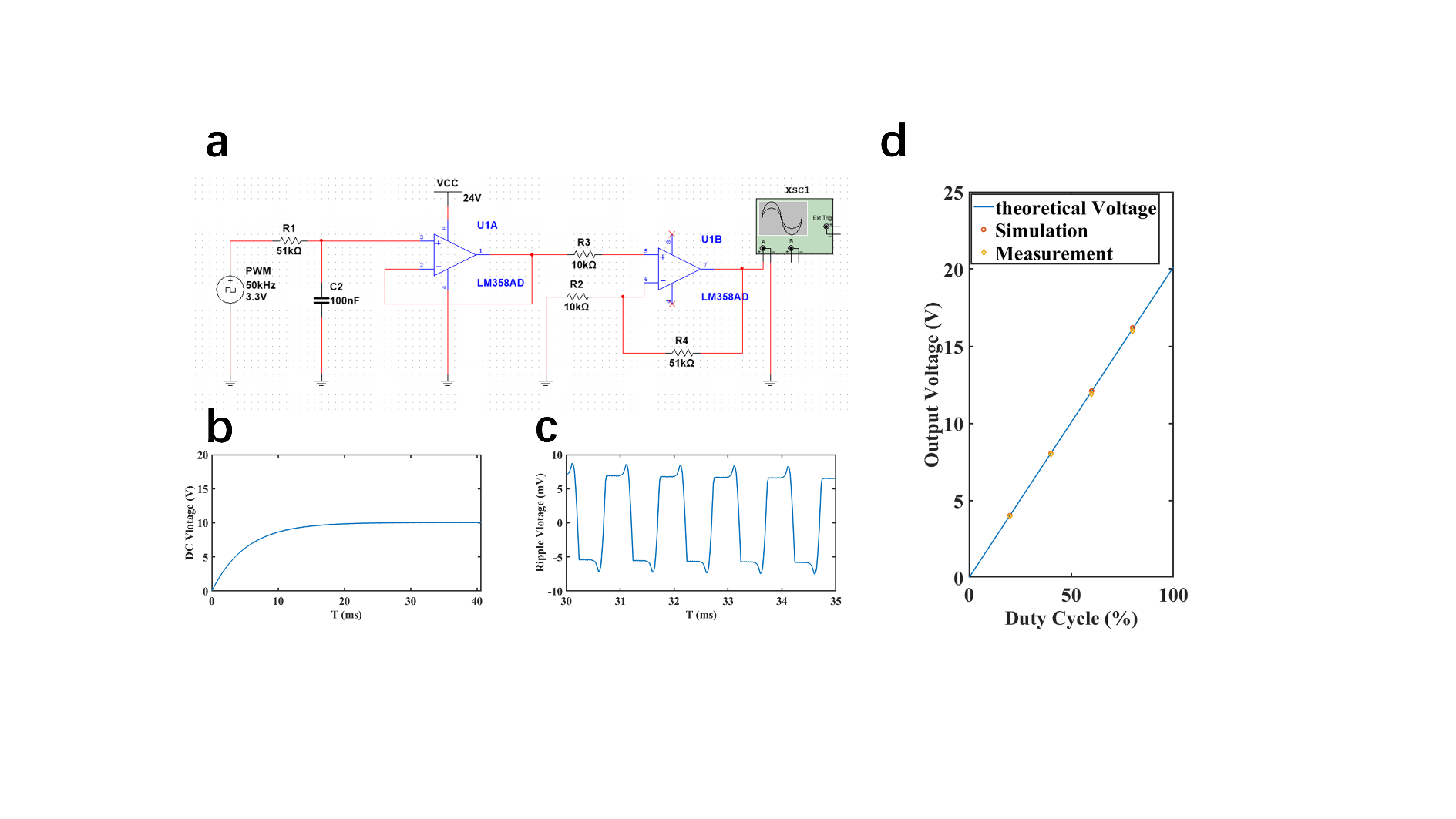


**Supplementary Fig. 2.** The structure and the performance of the PWM to DC control Voltage conversion circuit. **a.** The detailed structure of the conversion circuit. **b.** The time-domain response of the proposed circuit. **c.** The ripple occurs when the output voltage reaches a steady state. **d.** The theoretical, simulated and measured output voltage under different duty cycles.

**Supplementary Note 3. Comparison of odd-mode OAM and even-mode OAM generation based on a 16×16 array.**

In the manuscript, we proposed the OAM beam with *l*=2, 4, 6 to demonstrate the performance and potential application of the proposed metasurface. In this manuscript, the meta-particle in the metasurface array is rectangular arrangement, resulting in worse performance in odd-level OAM beam generation (including *l*=±1, ±3, ±5…). Therefore, compared with the even-level OAM (*l*=±2, ±4, ±6…), both the phase distribution and focusing efficiency of the odd-level OAM are relatively poor. To visually demonstrate this phenomenon, we conducted first-order and second-order OAM beam simulations on a 16×16 array. The simulation results are shown in **Supplementary Fig. 3.** Furthermore, we calculated both focusing efficiency and mode purity of the OAM with *l*=1 and *l*=2 and listed the results in Supplementary Table 1.

Theoretically, with a lower OAM level, the performance of the proposed metasurface will be better. However, when generating OAM waves with *l*=1, there are two phase vortex centers on the focusing plane, and thus, reducing the focusing efficiency and mode purity. With the increase in odd OAM mode, the vortex center on the focusing plane will also increase, and therefore, the focusing efficiency and mode purity will decrease rapidly, resulting in nearly chaotic amplitude and phase distribution in the odd mode OAM generation. However, for the even mode OAM, there is only one focusing center and vortex center in the focusing plane; therefore, maintaining a high mode purity in OAM generation even if *l*=6.

**
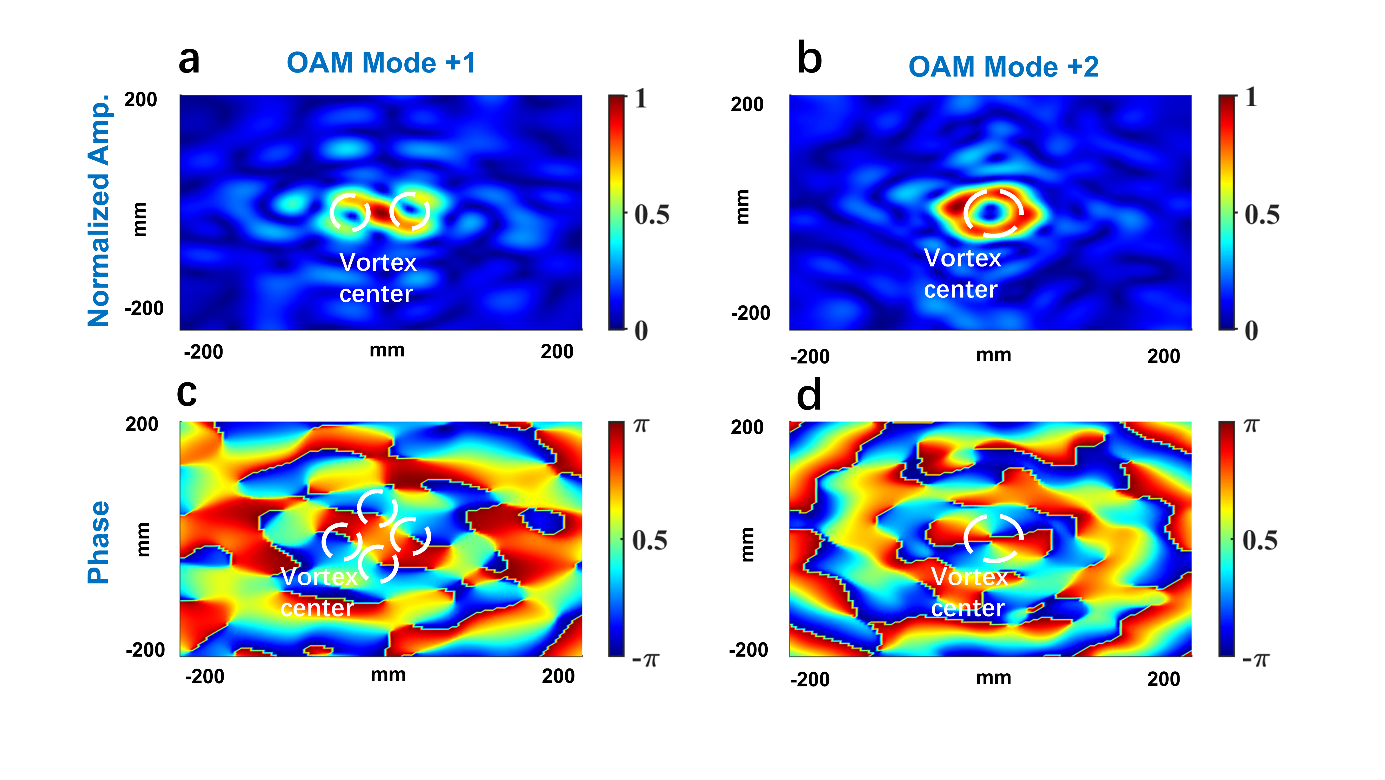
**

**Supplementary Fig. 3.** **a-b.** The normalized amplitude of the OAM wave in the plane *z*=200mm when *l*=+1 and +2. **c-d.** The phase distribution of the OAM wave in the plane *z*=200mm when *l*=+1 and +2.

**Supplementary table.1** Numerical analysis on OAM generation

| OAM Level | Mode Purity | Focusing efficiency |
| --- | --- | --- |
| 1 | 89.2% | 56.5% |
| 2 | 95.2% | 69.5% |

**Supplementary Note 4. Comparison of performance between a 16×16 array and an 8×8 array**

The simulation and measurement of the proposed metasurface in the manuscript are based on a 16×16 array and an 8×8 array, respectively. A smaller array will lead to a decrease in focusing efficiency, imaging resolution, and OAM mode purity. To directly show the influence of a smaller array, we conduct a simulation in OAM generation based on a 16×16 array and an 8×8 array. In the simulation, we generated OAM waves with *l*=2 in the plane *z*=200mm through a 16×16 array and an 8×8 array, and the corresponding results are shown in **Supplementary Fig. 3.** The numerical analysis of the simulation results is listed in Supplementary Table 2. With a smaller array scale, the mode purity and focusing efficiency decrease, and the focus area increases. The results show that though the performance of the metasurface deteriorates, the metasurfaces can still achieve the expected functions with the decrease in the array scale.


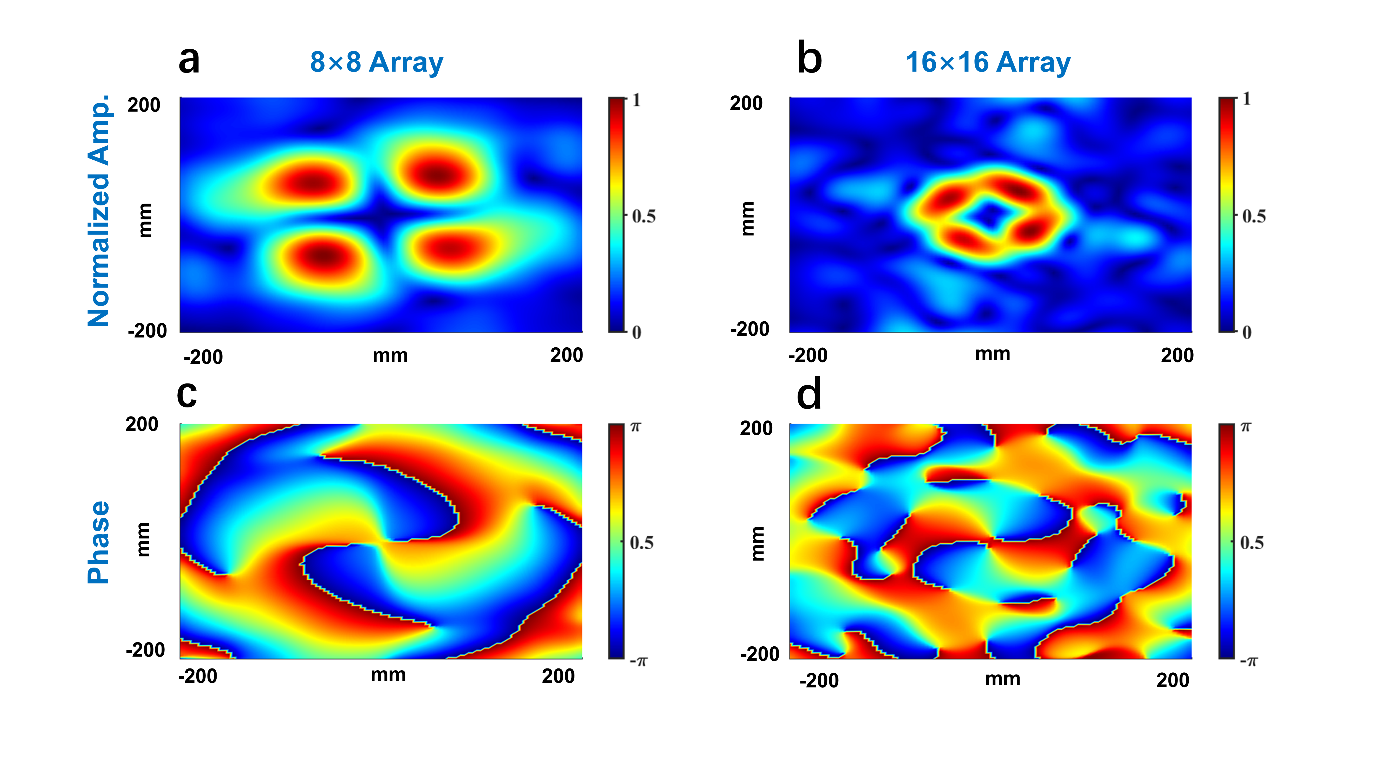


**Supplementary Fig. 4** **a-b.** The normalized amplitude of the OAM wave in the plane *z*=300mm when *l*=+2 with a 16×16 array and an 8×8 array. **c-d.** The phase distribution of the OAM wave in the plane *z*=300mm when *l*=+2 with a 16×16 array and an 8×8 array.

**Supplementary table.2** Numerical analysis on OAM generation

| Array scale | Mode Purity | Focusing efficiency |
| --- | --- | --- |
| 16×16 | 94.4% | 68.2% |
| 8×8 | 79.2% | 60.5% |
